# Effect of Hypoxia on *Ldh-c* Expression in Somatic Cells of Plateau Pika

**DOI:** 10.3390/ijerph13080773

**Published:** 2016-08-01

**Authors:** Dengbang Wei, Linna Wei, Xiao Li, Yang Wang, Lian Wei

**Affiliations:** 1 State Key Laboratory of Plateau Ecology and Agriculture, Qinghai University, Xining 10743, China; 2 College of Eco-Environmental Engineering, Qinghai University, Xining 10743, China; weilinna92@163.com (L.W.); lixiao2234566@163.com (X.L.); yangwangmd@163.com (Y.W.); weilian5318@163.com (L.W.)

**Keywords:** *Ldh-c*, plateau pika (*Ochotona curzoniae*), hypoxia, altitude, Qinghai-Tibetan Plateau

## Abstract

Sperm specific lactate dehydrogenases (LDH-C_4_) is a lactate dehydrogenase that catalyzes the conversion of pyruvate to lactate. In mammals, *Ldh-c* was originally thought to be expressed only in testes and spermatozoa. Plateau pika (*Ochotona curzoniae*), which belongs to the genus Ochotona of the Ochotonidea family, is a hypoxia-tolerant mammal living 3000–5000 m above sea level on the Qinghai-Tibet Plateau, an environment which is strongly hypoxic. *Ldh-c* is expressed not only in testes and sperm, but also in the somatic tissues of plateau pika. To reveal the effect of hypoxia on pika *Ldh-c* expression, we investigated the mRNA and protein level of *Ldh-c* as well as the biochemical index of anaerobic glycolysis in pika somatic tissues at the altitudes of 2200 m, 3200 m and 3900 m. Our results showed that mRNA and protein expression levels of *Ldh-c* in the tissues of pika’s heart, liver, brain and skeletal muscle were increased significantly from 2200 m to 3200 m, but had no difference from 3200 m to 3900 m; the activities of LDH and the contents of lactate showed no difference from 2200 m to 3200 m, but were increased significantly from 3200 m to 3900 m. Hypoxia up-regulated and maintained the expression levels of *Ldh-c* in the pika somatic cells. Under the hypoxia condition, plateau pikas increased anaerobic glycolysis in somatic cells by LDH-C_4_, and that may have reduced their dependence on oxygen and enhanced their adaptation to the hypoxic environment.

## 1. Introduction

The Qinghai-Tibet Plateau, with an average elevation of over 3000 m above sea level, is the highest and largest plateau on earth and it possesses a unique environment of nature and geography. Hypoxia is the most obvious climate characteristic on the plateau, in general; the air content decreases with the elevation at a rate of 0.67 kPa for 100 m, and the average oxygen content on the Qinghai-Tibet Plateau is only 60% of that at sea level. The oxygen concentration from the atmosphere to the tissues and cells reaches the cellular level to impact the basic units of life [1]. Therefore, over long-term evolution, many plateau-native animals have developed their own unique mechanisms to adapt to the hypoxic environment on the plateau.

Plateau pika (*Ochotona curzoniae*), an endemic species of the Qinghai-Tibet Plateau, inhabit meadows at altitudes 3000–5000 m above sea level. The pika is a key species in that it plays an important role in the biodiversity of the ecosystem on the Qinghai-Tibetan Plateau [2,3]. So far, pika fossil samples found on the north edge of the Qinghai-Tibet Plateau are about 37 million years old [4]. During evolution, the pika underwent a series of changes to adapt to the harsh environment. First, the pika obtains oxygen sufficiently by a special pulmonary structure and erythrocyte characteristics to get a high oxygen uptake capacity [5,6,7,8,9]. Secondly, the pika transports oxygen effectively by a large heart volume and a special cardiac structure to get a strong cardiac pumping function [10]. Thirdly, the pika has a high ratio of oxygen utilization by increasing the capillary and mitochondrial densities [11] and myoglobin (Mb) content [7,10] in tissues to get higher oxygen saturation in the arterial blood. Besides these physiological mechanisms, the pika reduces its dependence on oxygen by increasing anaerobic glycolysis in the skeletal muscle [12] and gluconeogenesis in the liver [13]. The molecular basis of these adaptations in pika has occurred with a series changes of genetic evolutionary and with genes related to hypoxia, such as HIF-1α [4,10,13,14,15,16,17,18,19,20,21]. We also found that *Ldh-c* (s testes-specific lactate dehydrogenase gene) is expressed not only in testes and sperm, but also in the somatic tissues of plateau pika [22].

The lactate dehydrogenase (LDH) family enzymes catalyze the inter-conversion of pyruvate to lactate with the concomitant oxidation/reduction of nicotinamide adenine dinucleotide hydrogen (NADH) to nicotinamide adenine dinucleotide (NAD^+^) [23]. Different forms of LDH are the products of three different genes: *Ldh-a*, *Ldh-b*, and *Ldh-c* which encode A, B and C subunits, respectively [24,25]. LDH consists of A and B subunits that assemble into homo- or hetero-tetramers that are distributed in the body in various combinations, reflecting the metabolic requirements of different tissues, and are consistent with the catalytic properties of the isozymes [26,27]. However, the homo-tetramer LDH-C_4_ was only detected in testes and spermatozoa and not in any other tissues or cells, and it was irreplaceable in the male reproductive capacity [28,29,30,31]. *Ldh-c* expressed in the somatic cells of plateau pika may play a crucial role in anaerobic glycolysis and ATP rapid generation; the pika has a reduced dependence on oxygen and an enhanced adaptation to the hypoxic environment due to increased anaerobic glycolysis by LDH-C_4_ in the somatic tissues [32].

Under hypoxia conditions, many organisms switch from aerobic metabolism to anaerobic metabolism in order to maintain their function. Since LDH is one of the main enzymes used under anaerobic conditions, studies have showed that hypoxic conditions resulted in the alteration of the LDH activity [33,34,35,36]. LDH activities in animals would increase after being exposed to hypoxic conditions [35,36]. LDH-A isozyme activity and its protein expression are increased most significantly at 24 h in cells under hypoxia. However, the LDH-B protein is unchanged while its mRNA is decreased [37]. The LDH activities in pika tissues had been proved to respond to the hypoxic environment, and the results showed the activities of LDH-A were up-regulated at a higher elevation [38]. However, up until now, the relationship between *Ldh-c* and hypoxia has not precisely been known.

In the current study, we investigated the effect of hypoxia on *Ldh-c* expression in somatic cells of plateau pika. The mRNA and protein level of pika *Ldh-c* was measured and the biochemical indexes of anaerobic glycolysis were assayed in different altitude conditions.

## 2. Material and Methods

### 2.1. Animals and Sample Collection

Plateau pikas were live-trapped from Qinghai Province in China. Pikas were divided into three groups: (1) high altitude (3900 m Group), pikas collected from Laji Mountain in Guide County at an altitude of 3900 m, the oxygen content is 174.2 g/m^3^; (2) medium altitude (3200 m Group), pikas collected from Haibei Alpine Meadow Ecosystem Research Station in Menyuan County at an altitude of 3200 m, the oxygen content is 196.6 g/m^3^; (3) low altitude (2200 m Group), pikas collected from Haibei Alpine Meadow Ecosystem Research Station, and raised in Xining City for 16 months, the oxygen content is 228.6 g/m^3^. The sample size is eight for each group above. In order to detect the mRNA levels of *Ldh-a* and *Ldh-b* of plateau pikas at different altitudes, the plateau pikas were live-trapped from Menyuan Country at an altitude of 3200 m, the oxygen content is 196.6 g/m³, and Guoluo Country at an altitude of 4200 m, the oxygen content is 164.6 g/m³, respectively, in Qinghai Province, China. The sample size is 10 for each group above. All animals were anesthetized with sodium pentobarbital (5%) and then sacrificed by cervical dislocation immediately before dissection. Heart, liver, brain and skeletal muscle were removed and stored in liquid nitrogen rapidly. All procedures involved in the handling and care of animals were in accordance with the China Practice for the Care and Use of Laboratory Animals and were approved by the China Zoological Society (permit number: GB 14923-2010).

### 2.2. RNA Extraction and Quantification of Ldh-c mRNA Level by qRT-PCR

Total RNA was isolated using TRIzol reagent (Invitrogen Corp., Carlsbad, CA, USA). RNA concentration and purity were assessed by Ultra Violet (UV) spectrophotometry (1.8 < A260/A280 < 2.0). RNA integrity was checked using electrophoresis. Reverse transcription reaction was carried out starting from 4 μg of total RNA using the First Strand complementary DNA (cDNA) Synthesis kit (Thermo Scientific, Boston, MA, USA).

To make standard curves, 1 μL of first-strand cDNA were amplified with Premix Ex Taq Version Kit (TaKaRa BIO INC., Kusatsu, Japan), and quantification of Polymerase Chain Reaction (PCR) products were used for plotting standard curves. The initial product concentration was set at 1 and standard curves were generated using a 10-fold serial dilution ranging from 1 to 10^−7^.

Quantitative Real-time Polymerase Chain Reaction (qRT-PCR) was performed using the SYBR^®^ Premix Ex Taq^™^ II (TaKaRa BIO INC., Kusatsu, Japan) protocol on BIO-RAD Connect real-time PCR detection system with cycling conditions of 95 °C for 3 min, followed by 40 cycles of 95 °C for 30 s and 60 °C for 30 s. *β*-actin was used as an internal control. The PCR primers for *Ldh-c*, *Ldh-a*, *Ldh-b*, GAPDH and *β*-actin were designed as follows: *Ldh-c*: forward, 5′-TATCGAGAATCTGATCGCAGAAGAC-3′ and reverse, 5′-GGGCAAGTTCATCAGCCAAATCC-3′, the amplicon length was 130 bp. *Ldh-a*: forward, 5′-TTGGTCCAGCGGAATGTA-3′ and reverse, 5′-GGTGAACTCCCAGCCTTT-3′, the amplicon length was 220 bp. *Ldh-b*: forward, 5′-TGTTGGACAAGTCGGAATG-3′ and reverse, 5′-CTGAAGAAACAGGCTCCC-3′, the amplicon length was 139 bp. *GAPDH*: forward, 5′-GGGAAATCGTGCGTGACTT-3′ and reverse, 5′-GCGGCAGTGGCCATCTC-3′, the amplicon length was 203 bp. *β*-actin: forward, 5′-CTCTTCCAGCCCTCCTTCTT-3′ and reverse, 5′-AGGTCCTTACGGATCTCCAC-3′, the amplicon length was 98 bp. *Ldh-c* mRNA level was normalized with *β*-actin mRNA to compensate for variations in initial RNA amounts. Normalization was carried out by dividing the logarithmic value of *Ldh-c* by the logarithmic value of *β*-actin. *Ldh-a* and *Ldh-b* mRNA level was normalized with *GAPDH* mRNA to compensate for variations in initial RNA amounts. Normalization was carried out by dividing the logarithmic value of *Ldh-a* and *Ldh-b* by the logarithmic value of *GAPDH*.

### 2.3. Western Blot Analysis

Total cellular proteins were extracted by RIPA buffer containing protein inhibitors, BCA protein assay kit (Pierce Biotechnology, Rockford, IL, USA) was used to assess protein concentration. Proteins were separated by sodium dodecyl sulfate polyacrylamide gel electropheresis (SDS-PAGE), and transferred onto a 0.22 mmol/L polyvinylidene difluoride (PVDF) membrane. After blocking the non-specific binding sites for 120 min with 5% non-fat milk, the membranes were incubated with a rabbit polyclonal antibody against LDH-C (SIGMA-ALDRICH, Saint Louis, MO, USA, at 1:4000 dilution) or GAPDH (Genetex, San Antonio, TX, USA, at 1:5000 dilution) at 4 °C overnight. The membranes were then washed with TBST (Tris-Buffered Saline with Tween-20) six times at room temperature for 10 min. After washing, the target protein was probed with the horseradish peroxidase (HRP)-conjugated goat anti-rabbit IgG antibody (Santa Cruz, Santa Cruz, CA, USA, at 1:6000 dilution) at 37 °C for 2 h. After 10 washes, the bound antibody was detected by chemiluminescence with the ECL Detection Reagent (Pierce Biotechnology, Rockford, IL, USA).

### 2.4. LDH Activities and LD Contents Assessment

The tissues were homogenized on ice as a 1:9 (w/v) dilution in 0.9% physiological saline. The homogenate was centrifuged at 2500 rpm/min at 4 °C for 10 min, and the supernatant was collected. Protein level was measured by Bradford assay. The LD assay kit and the LDH activity assay kit (Nanjing Jiancheng Bioengineering Institute, Nanjing China) were used to assess the LD content and LDH activity in tissues.

### 2.5. Data Analysis

Data were expressed as mean ± SD. Statistical analysis was performed by one-way analysis of ANOVA and Duncan’s test using SPSS 17.0 (SPSS Inc., Chicago, IL, USA). *p* < 0.05 was considered statistically significant.

## 3. Results

### 3.1. qRT-PCR Analysis of Ldh-a and Ldh-b mRNA Expression

The mRNA levels of *Ldh-a* and *Ldh-b* of plateau pikas at different altitudes were examined in the heart, liver, brain and skeletal muscle by qRT-PCR assays. As the statistics results showed in Table 1, the relative expression levels of *Ldh-a* and *Ldh-b* mRNA in the heart, liver, brain and skeletal muscle have no increase in plateau pika tissues with increasing of elevations from 3200 m to 4200 m.

### 3.2. qRT-PCR Analysis of Ldh-c mRNA Expression

**Figure 1:**
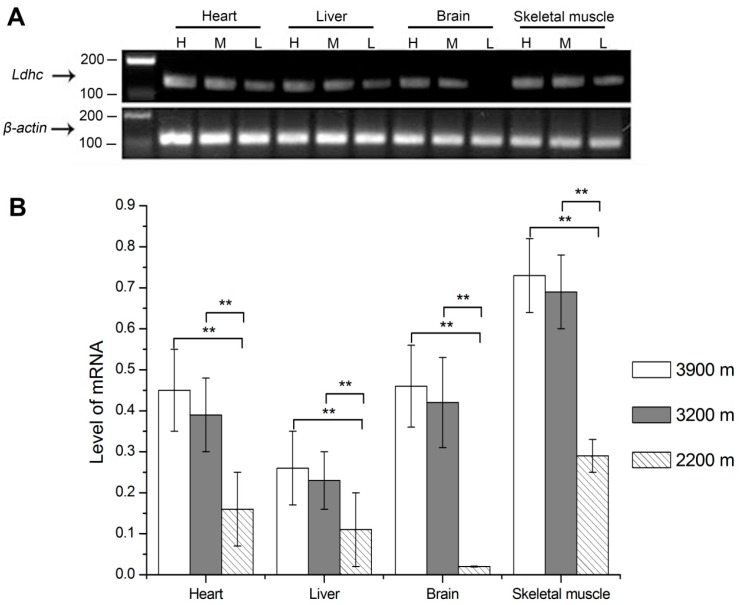
Quantification of *Ldh-c* mRNA levels in plateau pikas tissues at different altitudes. (**A**) The electrophoresis results of real-time PCR of *Ldh-c* and *β-actin* in plateau pika tissues; (**B**) Quantification of *Ldh-c* mRNA levels in plateau pikas tissues at different altitudes. H: high altitude group, 3900 m; M: medium altitude group, 3200 m; L: low altitude group, collected from Haibei Station, raised in Xining (2200 m) for 16 months. ** *p* < 0.01. The sample size is eight for each group.

The mRNA levels of *Ldh-c* of plateau pikas at different altitudes were examined in the heart, liver, brain and skeletal muscle by qRT-PCR assays. As the statistics results showed in Figure 1, the relative expression levels of *Ldh-c* at 3900 m, 3200 m and 2200 m altitude were 0.45 ± 0.10, 0.39 ± 0.09 and 0.16 ± 0.09 in the heart; 0.26 ± 0.09, 0.23 ± 0.07 and 0.11 ± 0.09 in the liver; 0.46 ± 0.10, 0.42 ± 0.11, and 0.02 ± 0.00 in the brain; and 0.73 ± 0.09, 0.69 ± 0.09 and 0.29 ± 0.04 in the skeletal muscle, respectively. For all tissues, the *Ldh-c* expression levels were significantly higher in the 3900 m group than in the 2200 m group; they were also significantly higher in the 3200 m group than in the 2200 m group (*p* < 0.01); however, there was no difference between the 3200 m and 3900 m groups.

### 3.3. Western Blotting Analysis of LDH-C Protein Expression

**Figure 2:**
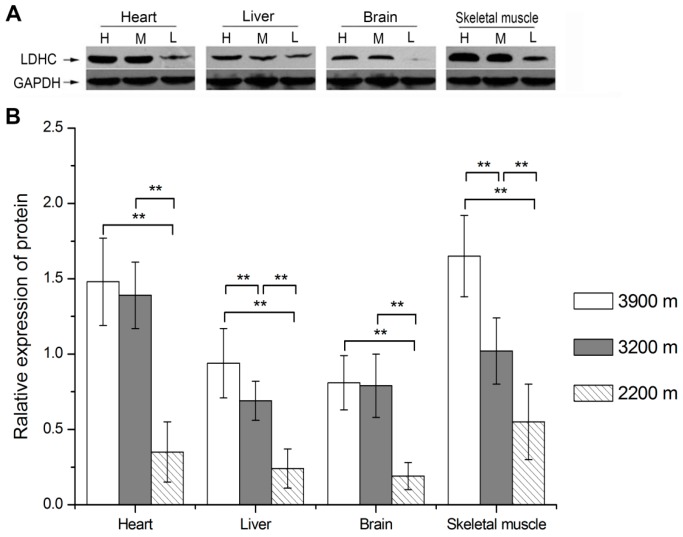
Quantification of LDH-C protein levels in plateau pika tissues at different altitudes. (**A**) Western blot result of LDH-C and GAPDH protein in plateau pika tissues; (**B**) Quantification of LDH-C protein levels in plateau pika tissues at different altitudes. H: high altitude group, 3900 m; M: medium altitude group, 3200 m; L: low altitude group, collected from Haibei Station, raised in Xining (2200 m) for 16 months. * *p* < 0.05, ** *p* < 0.01. The sample size is eight for each group.

The protein levels of LDH-C of plateau pikas at different altitudes were examined in the heart, liver, brain and skeletal muscle by Western blotting. The relative expression levels of LDH-C protein at the elevations of 3900 m, 3200 m and 2200 m were 1.48 ± 0.29, 1.39 ± 0.22 and 0.35 ± 0.20 in the heart; 0.94 ± 0.23, 0.69 ± 0.13 and 0.24 ± 0.13 in the liver; 0.81 ± 0.18, 0.79 ± 0.21 and 0.19 ± 0.09 in the brain; and 1.65 ± 0.27, 1.02 ± 0.22 and 0.55 ± 0.25 in the skeletal muscle, respectively. Statistics results showed that LDH-C expression levels increased with the altitude. It was significantly higher in the 3900 m group and the 3200 m group compared with the 2200 m group in the heart, liver, brain and skeletal muscle (*p* < 0.01). It was also significantly higher in the 3900 m group than in the 3200 m group in the liver (*p* < 0.05) and skeletal muscle (*p* < 0.01) (Figure 2).

### 3.4. LDH Activities and LD Contents in Plateau Pika Tissues at Different Altitudes

**Figure 3:**
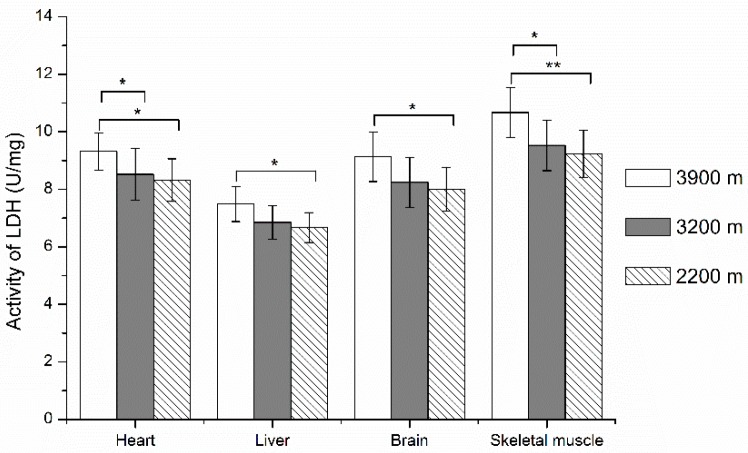
LDH activities in plateau pika tissues at different altitudes. For LDH activities at 3900 m, 3200 m, 2200 m group pikas, they were 9.32 ± 0.65, 8.52 ± 0.91 and 8.32 ± 0.74 in heart; 7.49 ± 0.61, 6.85 ± 0.59 and 6.67 ± 0.52 in liver; 9.13 ± 0.86, 8.24 ± 0.87 and 8.01 ± 0.76 in brain; 10.67 ± 0.87, 9.53 ± 0.88 and 9.24 ± 0.82 in skeletal muscle. * *p* < 0.05, ** *p* < 0.01. The sample size is eight for each group.

**Figure 4:**
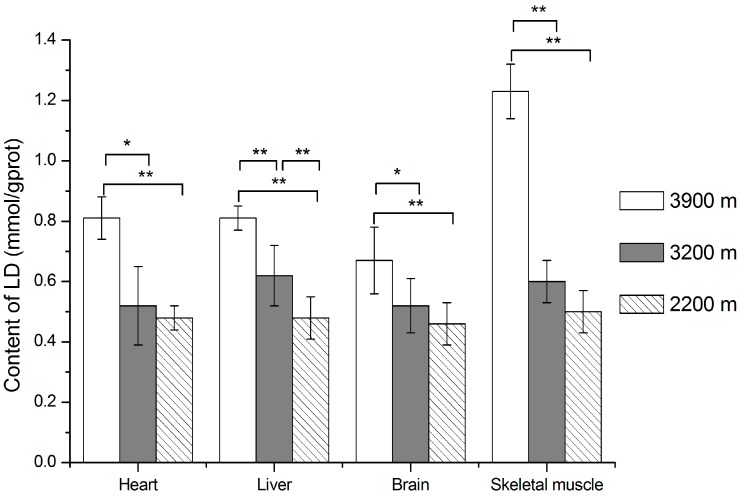
LD contents in plateau pika tissues at different altitudes. For LD contents of 3900 m, 3200 m, 2200 m group pikas, they were 0.81 ± 0.07, 0.52 ± 0.13 and 0.48 ± 0.04 in heart; 0.81 ± 0.04, 0.62 ± 0.10 and 0.48 ± 0.07 in liver; 0.67 ± 0.11, 0.52 ± 0.09 and 0.46 ± 0.07 in brain; 1.23 ± 0.09, 0.60 ± 0.07 and 0.50 ± 0.07 in skeletal muscle. * *p* < 0.05, ** *p* < 0.01. The sample size is eight for each group.

As shown in Figure 3 and Figure 4, LDH activities and LD contents increased with the altitude ascended from 2200 m to 3900 m. LDH activities were significantly higher in the 3900 m group than in the 2200 m group in pika heart, liver and brain (*p* < 0.05), and skeletal muscle (*p* < 0.01). They were also significantly higher in the 3900 m group than in the 3200 m group in heart and skeletal muscle (*p* < 0.05). For LD contents in tissues, it was significantly higher in the 3900 m group compared with the 3200 m group and 2200 m group in all tissues (*p* < 0.01); there was no difference between the 3200 m group and 2200 m group, except for the liver.

## 4. Discussion

The LDH family is encoded by three genes, *Ldh-a*, *Ldh-b*, and *Ldh-c*, for the expression of the LDH subunits LDH-A, LDH-B, and LDH-C. LDH-A is the most abundant in the liver and skeletal muscle and LDH-B is the most abundant in the heart and red blood cells [39]. The isoform LDH-A preferentially converts pyruvate to lactate and it is found predominantly in poorly vascularized tissues with low oxygen [39]. However, the isoform LDH-B is more active in aerobic conditions, converting lactate to pyruvate in well-oxygenated tissues [39]. Besides, *Ldh-c* was previously only detected being expressed in the testes and spermatozoa of mammals and birds, supporting its role in energy production in spermatids that favor lactate as a substrate with a characteristic aerobic glycolytic path to generate ATP [40]. LDH-C_4_ is the main LDH isozyme in sperm that accounts for 80%–100% of the total LDH activity in mammal spermatozoa [41].

Studies had demonstrated that the disruption of *Ldh-c* or the inhibition of LDH-C_4_ in sperm led to a rapid decline in sperm ATP levels [42,43], a decrease in progressive motility, and a failure to develop hyperactivated motility. Metabolic tracing experiments revealed that all consumed ^13^C-labeled pyruvate added in sperm culture medium was converted to lactate rather than oxidized in the tricarboxylic acid cycle. The ATP concentration was increased by more than 50% in the presence of exogenous pyruvate [44]. When carbonyl cyanide mchlorophenylhydrazone (CCCP) and NaCN were applied to suppress the oxidative phosphorylation in mitochondria, the vigorous motility of sperm was maintained and the amount of ATP was kept at the level equivalent to that without CCCP [44,45]. These results had proved that LDH-C_4_ is the key factor of sperm glycolysis, which has an important role in providing the ATP required for sperm motility and capacitation [42,45,46].

Surprisingly, we found that *Ldh-c* is expressed not only in testes and sperm, but it was also expressed generally in somatic tissues of plateau pika in our previous study [22]. Compared with LDH-A_4_ and LDH-B_4_, LDH-C_4_ had a lower *K*m for pyruvate (~0.052 mmol/L) and a higher *K*m for lactate (~4.934 mmol/L); thus, LDH-C_4_ was less sensitive to lactate inhibition than LDH-A_4_ and LDH-B_4_ [47]. These properties of pika LDH-C_4_ were beneficial for catalyzing the conversion of pyruvate to lactate even at a high concentration of lactate. Furthermore, both the activity of pika LDH-C_4_ inhibited by the specific inhibitor and the content of LDH-C_4_ reduced by silencing *Ldh-c* can also decrease the level of anaerobic glycolysis, ultimately leading to a decline in their exercise tolerance in hypoxic environments [32]. *Ldh-c* expressed in the somatic cells of plateau pikas enhances their anaerobic glycolysis, and generates ATP and lactate more rapidly. Lactate accumulation in the body can cause acidosis [48]. The liver is the main organ responsible for metabolizing excess lactate; in the liver, lactate is converted into pyruvate by LDH, and some of the pyruvic acid is oxidized through Kreb’s cycle, most of which is then converted into glucose and glycogen through gluconeogenesis [49]. LDH and pyruvate carboxylase (PC) are key enzymes in the process of gluconeogenesis; indeed, the rate of gluconeogenesis in the mammalian liver is related to the relative constituents of LDH isoenzymes and its enzymatic activities, as well as the enzymatic activity of PC, which is a rate-limiting enzyme gluconeogenesis [50]. During long-term evolution, the pikas evolved a series of physiological adaptations that allow them to convert lactate promptly in the liver. Our previous results indicated that, in the liver of plateau pikas, the mRNA levels of LDH-A, LDH-B and PC, the concentration of lactate, as well as the enzymatic activities of LDH and PC were significantly higher than those of plateau zokors [13]. Furthermore, the isoenzymatic spectrum of LDH in the livers of these two species showed that the main LDH isoenzymes in the liver of plateau pikas were LDH-A_3_B, LDH-A_2_B_2_, LDH-AB_3_ and LDH-B_4_, while LDH-A_4_ was the only isoenzyme found in the liver of mouse, rats and plateau zokors [13,51]. These results suggest that plateau pikas can rapidly convert the lactate produced from their skeletal muscle and other tissues by increasing the hepatic enzymatic activity of PC and their LDH, which contains a higher ratio of LDH-B subunits. Therefore, higher levels gluconeogenesis in the liver is one of the important mechanisms of plateau pikas’ adaptation to the hypoxic environment.

To reveal the role and physiological mechanism of LDH-C_4_ in the skeletal muscle of plateau pikas, we investigated the effect of silencing *Ldh-c* by RNAi on exercise tolerance as well as the physiological mechanism. We found that the expression levels of *Ldh-a* and *Ldh-b* have no statistical difference in skeletal muscle. Compared with the control group, *Ldh*-*c* mRNA and protein in the siRNA-*Ldh-c* group decreased by 82.18% and 82.29%, respectively; the LDH activity, LD content, and ATP level in the siRNA-*Ldh-c* group was reduced by 28.21%, 48.38% and 27.88%, respectively. Our results suggested that at least 27.88% of the ATP in pika skeletal muscle is catalyzed by LDH-C_4_ by anaerobic glycolysis; thus, pika has a reduced dependence on oxygen and enhanced adaptation to hypoxic environments due to increased anaerobic glycolysis by LDH-C_4_ in the skeletal muscle (unpublished). In order to shed light on the effect of LDH-C_4_ on the anaerobic glycolysis in plateau pika heart, liver, brain and skeletal muscle, we used a specific LDH-C_4_ inhibitor (*N*-isopropyl oxamate), and injected 1 mL 1 mol/L *N*-isopropyl oxamate into the biceps femoris muscle of the hind legs of plateau pika; each leg received 500 μL. The pikas of the control group were injected with the same volume of normal saline (0.9% NaCl). The results showed, compared to the control group, that the inhibition rates of *N*-isopropyl oxamate to LDH, LD and ATP were 31.98%, 20.90% and 28.70% in the heart [52]; 30.19% ± 3.90%, 32.22% ± 8.70% and 24.94% ± 7.80% in the liver [53]; 30.78%, 46.47% and 21.04% in the brain [54]; 37.12%, 66.27% and 32.42% in the skeletal muscle, respectively [32]. The above results suggested that pikas adapted to hypoxic environments by increasing anaerobic glycolysis by LDH-C_4_ in the somatic tissues.

Under hypoxia conditions, LDH-A and LDH-B subunits in the tissues of plain animals were increased significantly with the decrease of PO_2_ [38]. However, the contents of LDH-A and LDH-B in the heart, liver, kidney and skeletal muscle of plateau pikas had no differences at the same area at altitudes of 3400 m to 5000 m [38]. When the pikas inhabiting the spot at an altitude of 3400 m were transferred to 2300 m, the contents of LDH-A and LDH-B in the heart, liver, and kidney had no differences, while those of LDH-A and LDH-B in the skeletal muscle were down-regulated about 14%, and the activities of LDH showed no differences [38]. These results indicated that the LDH-A and LDH-B expression patterns and levels in plateau pikas had obvious differences from the plain animals; the pikas’ LDH-A was less sensitive to the changes of PO_2_, except for in the skeletal muscle. Our present study results showed that in the heart, liver, brain and skeletal muscle of plateau pikas, the mRNA expression levels of *Ldh-a* and *Ldh-b* had no increase in plateau pika tissues with increasing of elevations from 3200 m to 4200 m; the mRNA and protein expression levels of *Ldh-c* had no difference from the spot at 3900 m elevation to 3200 m elevation; and when the pikas inhabiting their original environment at an elevation of 3200 m were transferred to 2200 m, the mRNA and protein expression levels of *Ldh-c* decreased by more than 50%, but the activities of LDH showed no difference. These results suggested that, in their original inhabiting environment from the elevation of 3200 m to 3900 m, *Ldh-c* was less sensitive to PO_2_ changes, and its expression levels showed no difference in the mRNA and protein, but the higher PO_2_ in the spot where the pika is a non-indigenous animal had a significant inhibition on the *Ldh-c* expression.

## 5. Conclusions

In conclusion, the expression of *Ldh-c* in the somatic tissues of plateau pikas is an important adaptation mechanism. In spite of lowering the PO_2_ in their original inhabiting environment with increasing altitudes, the expression levels of *Ldh-c* in the pikas were not up-regulated by the lower PO_2_, but it was thought to be one of the important factors that maintained the expression levels of *Ldh-c* in the somatic tissues of plateau pikas. Under hypoxia conditions, plateau pikas increased the anaerobic glycolysis in somatic cells by LDH-C_4_, and that may reduce their dependence on oxygen and enhance their adaptation to the hypoxic environment.

## Figures and Tables

**Table 1:** Quantification mRNA levels of *Ldh-a*, *Ldh-b* in plateau pika tissues at 3200 m and 4200 m altitudes.

Tissues	*Ldh-a*		*Ldh-b*	
	3200 m	4200 m	3200 m	4200 m
heart	0.955 ± 0.029 **	0.811 ± 0.045	0.973 ± 0.022 **	0.880 ± 0.024
liver	0.959 ± 0.087	0.895 ± 0.095	0.909 ± 0.037	0.894 ± 0.035
brain	0.919 ± 0.032 **	0.785 ± 0.033	0.870 ± 0.048 **	0.785 ± 0.033
skeletal muscle	0.935 ± 0.037	0.847 ± 0.170	0.735 ± 0.046 *	0.641 ± 0.118

* *p* < 0.05, ** *p* < 0.01; The sample size is 10 for each group.
